# Supplementary material for: Dynamic transcriptomic profiles of zebrafish gills in response to zinc supplementation
Source: BMC Genomics. 2010 Oct 11;11:553. doi: 10.1186/1471-2164-11-553 (PMC3091702; doi:10.1186/1471-2164-11-553)
Supplement: Additional file 2 — Interactive Direct Interaction Network representing the molecular interactions between zinc, copper, iron, calcium and proteins encoded by transcripts changed by zinc supplementation. Mini web-site containing index.html and hyperlinked pages in subdirectory describing a Direct Interaction Network automatically generated based on curated interactions contained within the proprietary PathwayArchitect database. Ovals represent proteins and the circles symbolize metal ions. Objects are coloured by their abundance in zebrafish at the time-point they were significantly different from the control is a scale from -4 fold (dark green) to +4 fold (dark red). Where significant differences were found at more than one time-point, the colour overlay shows expression at the first instance. Dark blue squares denote 'binding', and light blue squares 'expression'; green squares stand for 'regulation', green diamonds for 'metabolism', and green circles for 'promoter binding'. Arrow heads indicate directionality of the interaction where annotated. All nodes and edges can be further interrogated by selecting the relative area of the image. [file 1471-2164-11-553-S2.zip › PathwayArchitect Zn xs DIN/688352.html]

# REGULATION:

|  |  |
| --- | --- |
| Type | REGULATION |
| Effect | Negative |


---

|  |  |
| --- | --- |
| Score | 0 |


---

|  |  |
| --- | --- |
| Reference Count | 6 |


---

|  |  |
| --- | --- |
| Mechanism | Unknown |


---

|  |  |
| --- | --- |
| Reference:0 || Sentence | "However, only in PD was there an increased total iron level, decreased ferritin content, decreased copper content, and an increased zinc concentration in substantia nigra." |
| PMID | 1832073 |
| Year | 1991 |
| Species | Human |
| Journal | Brain |
| RefScore | 2 |
| Source | PArchNLP |
  |
|  |  |


---

|  |  |
| --- | --- |
 Reference:1 || Sentence | "Twelve JCA patients had reduced serum iron (Fe), 6 reduced serum zinc (SZn), 14 reduced intra-erythrocytic zinc (EZn) and 2 reduced serum copper (SCu)." |
| PMID | 8652951 |
| Year | 1995 |
| Species | Human |
| Journal | Acta Paediatr |
| RefScore | 1 |
| Source | PArchNLP |
  ||  |  |


---

|  |  |
| --- | --- |
 Reference:2 || Sentence | "Levels of circulating ceruloplasmin (measured by rocket immunoelectrophoresis) were not altered by iron deficiency, although p-phenylenediamine oxidase activity and plasma copper were reduced approximately 30%." |
| PMID | 11880554 |
| Year | 2002 |
| Species | Rat |
| Journal | J Nutr |
| RefScore | 2 |
| Source | PArchNLP |
  ||  |  |


---

|  |  |
| --- | --- |
 Reference:3 || Sentence | "CONCLUSIONS: Non-anemic iron depletion characterized by low iron stores is associated with negative impacts on copper status." |
| PMID | 12480800 |
| Year | 2002 |
| Species | Human |
| Journal | J Am Coll Nutr |
| RefScore | 3 |
| Source | PArchNLP |
  ||  |  |


---

|  |  |
| --- | --- |
 Reference:4 || Sentence | "When dietary copper was 2.0 mg/kg of diet, high iron decreased (p<0.008) cardiac and hepatic copper, plasma copper and ceruloplasmin, and increased (p<0.02) cardiac weight, hepatic iron and plasma cholesterol." |
| PMID | 11396784 |
| Year | 2001 |
| Species | Rat |
| Journal | J Trace Elem Med Biol |
| RefScore | 1 |
| Source | PArchNLP |
  ||  |  |


---

|  |  |
| --- | --- |
 Reference:5 || Sentence | It is clear from this study that the changes in copper levels that accompany iron deficiency are not mediated by changes in transcription of the genes involved in copper transport. |
| Year | 2004 |
| PMID | 15157943 |
| Species | Human |
|  | Rat |
| Journal | J Nutr Biochem |
| RefScore | 2 |
| Source | PArchNLP |
  |


---

|  |  |
| --- | --- |
